# Identification of gene mutations in six Chinese patients with maple syrup urine disease

**DOI:** 10.3389/fgene.2023.1132364

**Published:** 2023-02-24

**Authors:** Lulu Li, Xinmei Mao, Nan Yang, Taoyun Ji, Shunan Wang, Yulan Ma, Haihe Yang, Yuting Sang, Jinqi Zhao, Lifei Gong, Yue Tang, Yuanyuan Kong

**Affiliations:** ^1^ Department of Newborn Screening Center, Beijing Obstetrics and Gynecology Hospital, Capital Medical University, Beijing Maternal and Child Healthcare Hospital, Beijing, China; ^2^ Peking University First Hospital Ningxia Women and Children’s Hospital (Ningxia Hui Autonomous Region Maternal and Child Health Hospital), Yinchuan, China

**Keywords:** maple syrup urine disease, high-throughput sequencing, gene mutation, neonatal screening, metabolic disorders

## Abstract

**Background:** Maple syrup urine disease (MSUD) is a rare autosomal recessive amino acid metabolic disease. This study is to identify the pathogenic genetic factors of six cases of MUSD and evaluates the application value of high-throughput sequencing technology in the early diagnosis of MUSD.

**Methods:** Clinical examination was carried out for patients and used blood tandem mass spectrometry (MS/MS), urine gas chromatography-mass spectrometry (GC/MS), and the application of high-throughput sequencing technology for detection. Validate candidate mutations by polymerase chain reaction (PCR)—Sanger sequencing technology. Bioinformatics software analyzed the variants’ pathogenicity. Using Swiss PDB Viewer software to predict the effect of mutation on the structure of BCKDHA and BCKDHB proteins.

**Result:** A total of six MSUD patients were diagnosed, including four males and two females. Nine variants were found in three genes of six MSUD families by high-throughput sequencing, including four missense mutations: c.659C>T(p.A220V), c.818C>T(p.T273I), c.1134C>G(p.D378E), and c.1006G>A(p.G336S); two non-sense mutations: c.1291C>T(p.R431*) and c.331C>T(p.R111*); three deletion mutations: c.550delT (p.S184Pfs*46), c.718delC (p.P240Lfs*14), and c.795delG (p.N266Tfs*64). Sanger sequencing’s results were consistent with the high-throughput sequencing. The bioinformatics software revealed that the mutations were harmful, and the prediction results of Swiss PDB Viewer suggest that variation affects protein conformation.

**Conclusion:** This study identified nine pathogenic variants in the *BCKDHA*, *BCKDHB*, and *DBT* genes in six MSUD families, including two novel pathogenic variants in the *BCKDHB* gene, which enriched the genetic mutational spectrum of the disease. High-throughput sequencing is essential for the MSUD’s differential diagnosis, early treatment, and prenatal diagnosis.

## Introduction

Maple syrup urine disease (MSUD, MIM248600), also known as branched-chain ketoaciduria, is an amino acid metabolic disorder named for the peculiar maple syrup odor of the urine of affected children, which was first reported in 1954 (MENKES et al., 1954). MSUD is a rare autosomal recessive metabolic disorder. The multi-enzyme complex functional defective of branched-chain α-keto acid dehydrogenase complex (BCKDC) in the cellular mitochondrial matrix will cause the accumulation of branched-chain amino acids (leucine, isoleucine, and valine) and the corresponding α-ketoacid derivatives (α-ketoisoacetic acid, α-keto-β-methylpentanoic acid, and α-ketoisovaleric acid) in brain tissue and body fluids (Strauss et al., 2006; Blackburn et al., 2017). The accumulation of branched-chain amino acids and the corresponding keto acid derivatives will produce neurotoxic effects on brain tissue, leading to severe brain developmental disorders and other neurological damage with a high lethality and disability rate (Wendel et al., 1983). Based on the time of onset, progression rate, and reactivity to vitamin B1, MSUD can be divided into Classic, Intermittent, Intermediate, Thiamine-responsive, and E3 deficiency (Yang et al., 2012; Douglas et al., 2020). Classic is the most common type and severe type in the neonatal period, with patients often dying within a few days. Intermediate MSUD can develop at any age and exhibits growth and intellectual development with milder symptoms than Classic. Patients with Intermittent MSUD present with intermittent seizures, asymptomatic during intervals. Patients’ growth and neurodevelopment are normal, and the Intermittent MSUD is similar to the Classic at the onset. Patients with Thiamine-responsive can improve their tolerance to leucine through thiamine supplementation, and their clinical manifestation is similar to the Intermediate MSUD. E3 deficiency is the rarest type of MSUD and is often associated with severe lactic acidemia and neurological impairment (Flaschker et al., 2007; Frazier et al., 2014; Fang et al., 2021)MSUD has significant racial and geographic variability, with a global incidence of 1/185,000 and a prevalence as high as 1/380 in the Mennonite population (Carleton et al., 2010). MSUD has a complex clinical presentation, with early manifestations mainly consisting of feeding difficulties, vomiting, lethargy, metabolic acidosis, and a peculiar maple sugar odor in urine and sweat during infancy (mainly in the early neonatal period). Later stages often progress to degenerative neuropathy, such as convulsions, hypoglycemia and increased muscle tone, which can lead to death in severe cases (Flaschker et al., 2007; Frazier et al., 2014). Due to the lack of specific clinical manifestations, MUSD is often misdiagnosed as neonatal encephalopathy, neonatal sepsis or other abnormal metabolic diseases. If the diagnosis and treatment are prompt, some children can reach normal intelligence scores. Therefore, the early diagnosis and treatment of MSUD are necessary (Medina et al., 2021).

This study analyzed the clinical data and genetic test results of six children with MSUD admitted to the Beijing Newborn Disease Screening Center and the Maternal and Peking University First Hospital Ningxia Women and Children’s Hospital (Ningxia Hui Autonomous Region Maternal and Child Health Hospital). Aim to explore the genetic etiology of MSUD and the value of genetic testing for the early diagnosis of MSUD and to enhance the acquaintance and diagnosis of this disease.

## Methods and materials

### Medical records and ethical sight

Case 1, male, full-term newborn infant, could not hold objects at four months, could not sit at six months, cried easily, and was slow to respond at two years old. The patient registered at the Beijing Newborn Disease Screening Center at 28 months for “slow response, slow learning, and inability to walk” (Table 1). The MS/MS and GS/MS detected elevated levels of leucine, isoleucine, and their corresponding α Ketoacid levels were elevated (Table 2). After diagnosis, the patient adhered to diet therapy and is now 16 years old, 180 cm tall, 94 kg, irritable, and mildly neurodevelopmentally delayed (Table 1).

**TABLE 1:** Clinical and genetic characteristics of patients recruited in this study.

Case	Gender	Ethnic group	Age	Age of onset	Clinical outcome	Clinical phenotype	Genetic characteristics					
							Gene	Zygote type	Allele origin	Variant location	Nucleotide (amino acid) change	Novel variant
1	male	Han	16 y7 m	28 m	Slight neurodevelopment delaydelay	Intermediate	*BCKDHA*	C-het	P	E6	c.659C>T(p.A220V)	N
									M	E6	c.795delG (p.N266Tfs*64)	N
2	male	Han	1 y	25 d	Normal neurodevelopment	Classic	*BCKDHB*	C-het	P	E10	c.1134C>G(p.D378E)	Y
									M	E7	c.818C>T(p.T273I)	N
3	male	Han	5 y3 m	6 d	Severe neurodevelopment delay	Classic	*BCKDHB*	C-het	P	E5	c.550delT (p.S184Pfs*46)	N
									M	E6	c.718delC (p.P240Lfs*14)	Y
4	female	Han	—	8 d	Died	Classic	*BCKDHB*	C-het	P	E3	c.331C>T(p.R111*)	N
									M	E9	c.1006G>A(p.G336S)	N
5	female	Hui	—	15 d	Died	Classic	*DBT*	hom	P/M	E11	c.1291C>T(p.R431*)	N
6	male	Hui	—	10 d	Died	Classic	*DBT*	hom	P/M	E11	c.1291C>T(p.R431*)	N

y, year; d, day; m, month; Y, yes; N, no; hom, homozygote; C-het, compound heterozygote; P, paternal; M, maternal; E, exon; I, intron.

**TABLE 2:** Test results of blood tandem mass spectrometry and gas chromatography-mass spectrometry.

Patient	Blood tandem mass spectrometry					Gas chromatography-mass spectrometry					
	LEU (70-330)	VAL (65-300)	VAL/PHE (1.2-4.8)	LEU/PHE (1.5-3.4)	LEU/ALA (0.1-0.99)	2-Keto-isovaleric (0-0.1)	2-Keto-3-methylvaleric (0)	2-Keto-isocaproic (0)	2-OH-isovaleric (0)	2-OH-3-methylvaleric (0)	2-OH-isocaproic (0)
1	772.01	486.2	8.98	14.25	3.87	11.14	28.19	27.55	73.19	0	3.37
2	1963.89	840.46	12.59	29.42	9.39	0	0	7.79	26.59	2.96	0
3	1633.40	486.68	52.24	52.25	44.89	12.90	23.12	25.68	32.74	23.09	—
4	4507.05	1028.41	20.61	90.32	31.72	140.2	198.2	374.3	246.8	15.2	—
5	2040.39	695.38	17.70	51.94	22.52	19.8	42.8	94.3	68.6	6.7	—
6	2760.98	672.40	16.43	67.47	28.55	—	—	—	—	—	—

Case 2, male, full-term delivered *via* cesarean section, 25 days of age, registered at the Beijing Newborn Screening Center for “elevated leucine and valine on neonatal diseases screening.” The MS/MS detected elevated leucine, isoleucine, and valine levels; the GC/MS showed increased 2-keto-isobaric acid, 2-hydroxyisovaleric acid, and 2-hydroxy-3-methyl valeric acid (Table 2). After diagnosis, the patient insisted on diet treatment. Nowadays, the patient is 11 months old, with a height of 72 cm, a weight of 9.8 kg, good spirits, and normal neural development (Table 1).

Case 3, male, full-term newborn infant, six days of age, registered at Beijing Children’s Hospital for “tic, convulsions, and drowsiness.” The patient had stable vital signs, drowsiness, poor response, and weakness cry. The MS/MS and GC/MS were used to detect leucine, isoleucine and the corresponding level of α-ketoacid is elevated (Table 2). Currently, the patient is five years old and treated intermittently, with poor growth and severe neurodevelopmental retardation (Table 1).

Case 4, female, full-term newborn infant, first diagnosis in Peking University First Hospital Ningxia Women and Children’s Hospital (Ningxia Hui Autonomous Region Maternal and Child Health Hospital) at eight days of age for “poor feeding, poor response, persistent irritability and crying.” The MS/MS and GC/MS were used to detect leucine, isoleucine, and the corresponding level of α-ketoacid evaluated (Table 2). The medical history revealed that the patient began to have the poor appetite at four days of age and gradually aggravated the symptoms of irritability. The patient had less response on the physical examination, limb hypertonia, and cranial magnetic resonance imaging revealed bilateral cerebellar hemispheres, bilateral internal capsule, symmetrically increased signal intensity within the subcortical aera of the central sulcus and the white matter of centrum ovale.

Case 5, female, full-term newborn infant, first diagnosis in Peking University First Hospital Ningxia Women and Children’s Hospital (Ningxia Hui Autonomous Region Maternal and Child Health Hospital) at 15 days of age with “poor appetite for 15 days and refusal of milk for one day”. The patient was found to be drowsiness, poor response, weak cry, and decreased muscle tone (hypotonia). The EEG revealed an abnormal signal.

Case 6, male, full-term newborn infant, first diagnosis in Peking University First Hospital Ningxia Women and Children’s Hospital (Ningxia Hui Autonomous Region Maternal and Child Health Hospital) at ten days of age with “poor appetite for seven days and drowsiness for two days.” The physical examination revealed stable vital signs, drowsiness, poor response, poor nutrition, and a weak cry. The EEG showed an abnormal signal, and the chest X-ray revealed a patchy hyperdensity shadow in both lungs, indicating infectious lesions in both lungs.

All the mentioned patient’s parents had normal phenotypes, not consanguineous marriage, and no family history of genetic or infectious disease. The Beijing Obstetrics Gynecology Hospital of Capital Medical University Ethics Committee approved this study (2022-KY-087-01), and all family members (or guardians) signed informed consent.

### Blood tandem mass spectrometry

The dry blood filter paper for neonatal screening was detected by Blood tandem mass spectrometry. Each specimen was taken 3 mm diameter of blood spots, using TQD tandem mass spectrometry (Triple Quadrupole Mass Spectrometry) (TQ Acquity Mass Spectrometer, Waters Corporation, United States). Use the NeoBase Non-derivatized MSMS Kit (Perkinelmer, Turku, Finland) and tandem mass spectrometry methods to detect amino acids and acylacrnitine with metabolic disorders.

### Gas chromatography-mass spectrometry

5–10 ml of fresh urine was collected from patients, and the urine was processed by removing urea, adding internal standards, removing protein, performing vacuum drying, and trimethylsilyl derivatization. Use gas chromatography-mass spectrometry (Shimadzu GCMS-QP2010) to analyze the treated urine to identify the components of organic acids in urine. After that, analyzed qualitatively, and quantitatively of the detection peaks.

### High throughput sequencing and bioinformatics analysis

The experimental procedure of high throughput sequencing was around as follows.

DNA extraction: The subject and their phenotypically normal parents would be extracted 5 ml of peripheral blood from the elbow vein which used the classical phenol-chloroform method. The peripheral blood samples are stored at −20°C for backup.

Genomic library preparation: Genomic DNA is randomly fragmented to a certain size, then add adopter primers to prepare the desired library.

Hybridization capture: Using GenCap gene sequence capture technology, the probe is fully mixed with the sample-related gene target region library to enrich the DNA sequences in the exon region.

Sequencing: Use High-throughput sequencing to process the quality-checked products.

Data filtering and analysis: The raw data are filtered and analyzed to assess the sequencing quality. Use human genome standard sequence hg19 (Genome Reference Consortium GRCh37, hg19) to compare with the filtered data and count and analyze the data such as single nucleotide polymorphisms (SNPs) and insertion-deletion mutations (InDels). Afterward, annotation and analysis of HGMD, ExAC, and other databases were performed to screen candidate pathogenic variants to comprehend whether the identified variants were new or already reported.

### Primer designing and mutation confirmation

According to the results of candidate pathogenic variants screened by high-throughput sequencing, BCKDHA(NM_000155), BCKDHB(NM_183050), and DBT(NM_001918) gene sequences were obtained from http://genome.ucsc.edu. The standard procedures would perform the PCR amplification using the Primer5.0 primer design tool to design specific PCR primers (Supplementary Table S1), the PCR amplification was performed according to standard procedures. The PCR products were purified and processed the DNA sequencing. The sequencing results were analyzed by CodonCode Aligner software, along with parental origin verification.

### Predictive analysis of pathogenicity

Functional prediction of missense mutant loci was performed using Sorting Intolerant From Tolerant (SIFT,http://www.sift.jcvi.org/), Polyphen-2 (http://genetics.bwh.harvard.edu/pph2), Mutation taster (http://www.mutationtaster.org/), and REVEL (https://sites.google.com/site/revelgenomics/). Variant frequencies were determined in the 1000 Genomes Project, ExAC (http://exac.broadinstitute.org/), and GnomAD ALL (http://gnomad-sg.org/) database. Finally, the American College of Medical Genetics and Genomics (ACMG) 2015 guidelines were used to interpret variants.

Used Swiss-PDB viewer software to predict the evaluation of the crystal structure of the mutant proteins. The protein structures of *BCKDHA* and *BCKDHB* (PDB ID:1DTW) were acquired from the PBD database and combined with Swiss PDB Viewer 4.1.0 software to visualize the protein structures and predict the effect of the mutation sites on the tertiary protein structures.

## Results

### Clinical characteristics

This study included six MSUD cases, including four males and two females—those patients among four Han people and two Hui people. Five patients had clinical manifestations of classic MUSD in the neonatal period, particularly with vomiting, lethargy, poor appetite, tic, and feeding difficulties, and one patient had an intermediate MSUD (Table 1). Six patients’ MS/MS revealed significantly elevated leucine, isoleucine, valine, and valine to phenylalanine ratios. GC/MS results were also significantly elevated in the other five patients, except case 6 (Table 2).

Case 4 had a high-signal lesion on cranial MRI, and cases 5 and 6 had abnormal EEG. Cases 1 and 2 received treatment and were followed up regularly with good compliance. Case 3 was treated intermittently with a poor prognosis. Cases 4, 5, and 6 died in the neonatal period (Table 1).

### Gene sequencing results

**FIGURE 1:**
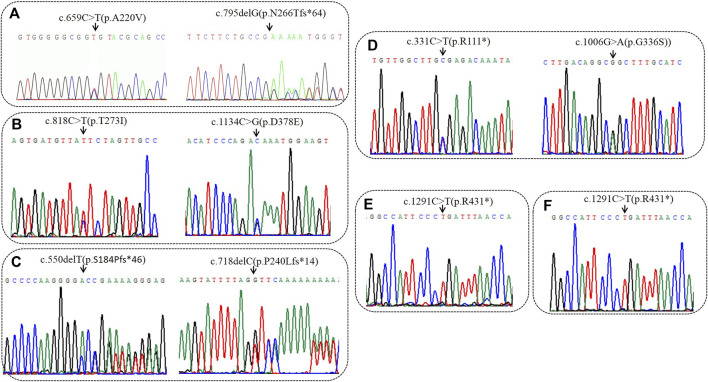
Sequencing analysis of all available members in six families with MSUD. **(A)** In family 1, sanger sequencing revealed the patient had the compound heterozygous variantis c659C>T(p.A220V) of paternal origin and c794delG (p.N266Tfs*64) of maternal origin in *BCKDHA*. **(B)** In family 2, sanger sequencing revealed the patient had the compound heterozygous variantis c1134C>G(p.D378E) of paternal origin and c818C>T(p.T273I) of maternal origin in *BCKDHB*. **(C)** In family 3, sanger sequencing revealed the patient had the compound heterozygous variantis c550delC (p.S184Pfs*46) of paternal origin and c718delC (p.P240Lfs*14) of maternal origin in *BCKDHB*. **(D)** In family 4, sanger sequencing revealed the patient had the compound heterozygous variantis c331C>T(p.R111*) of paternal origin and c1006G>A(p.G336S) of maternal origin in *BCKDHB*. **(E)** In family 5, the patient was homozygous of c1291C>T(p.R431*) in *DBT.* **(F)** In family 6, the patient was homozygous of c1291C>T(p.R431*) in *DBT.* Black arrows point to the mutation sites.

High-throughput results revealed that case 1 carried compound heterozygous mutations c.659C>T(p.A220V) and c.795delG (p.N266Tfs*64) of BCKDHA gene (Figure 1A). Sanger sequencing confirmed that the c.659C>T(p.A220V) mutation was inherited from the father with the normal phenotype, and the c.795delG (p.N266Tfs*64) mutation was inherited from the mother with the normal phenotype. Case 2 carried the compound heterozygous mutations c.818C>T (p.T273I) and c.1134C>G (p.D378E) of the *BCKDHB* gene (Figure 1B). Sanger sequencing confirmed that the c.818C>T (p.T273I) mutation was inherited from the mother with the normal phenotype, and the c.1134C>G (p.D378E) mutation was inherited from the father with the normal phenotype. Case 3 carried the compound heterozygous mutations c.550delT (p.S184Pfs*46) and c.718delC (p.P240Lfs*14) of the *BCKDHB* gene (Figure 1C). Sanger sequencing confirmed c.550delT (p.S184Pfs*46) mutation is inherited from the father with the normal phenotype, c.718delC (p.P240Lfs*14) mutation was inherited from the mother with the normal phenotype. Case 4 carried the *BCKDHB* gene compound heterozygous mutation c.331C>T (p.R111*) and c.1006G>A (p.G336S) (Figure 1D). Sanger sequencing confirmed that the c.331C>T (p.R111*) mutation was inherited from the father with normal phenotype, and the c.1006G>A (p.G336S) mutation was inherited from the mother with normal phenotype. Cases 5 and 6 carry homozygous mutation c.1291C>T (p.R431*) of *DBT* gene (Figures 1E, F). Their parents are heterozygous carriers of c.1291C>T (p.R431*). Six families conform to autosomal recessive inheritance.

### Pathogenicity prediction analysis

Variation c.1134C>G (p.D378E) and c.718delC (p.P240Lfs*14) are two novel mutations that have not been reported (Table 1), and 1000 Genomes Project, ExAC database, and GnomAD database have not included these two mutations. We used Bioinformatics software analysis to analyze variation c.1134C>G (p.D378E): the predicted score of SIFT software was 0.021, the predicted score of Polyphen-2 software was 0.663, the predicted value of Mutation Taster software was 1, and the REVEL score was 0.787. All the mentioned software indicated that the variation was pathogenic. Other missense mutations indicated as pathogenic were analyzed jointly by SIFT, Polyphen-2, Mutation Taster software, and REVEL (Table 3). Meanwhile, all the mutation-related diseases were highly consistent with the patient’s clinical phenotype, which was the supporting pathogenic evidence.

**TABLE 3:** Bioinfomatic analysis of missense variants.

	ExAC ALL	GnomAD ALL	SIFT	Polyphen2	MutationTaster	REVEL	ACMG
c.659C>T(p.A220V)	0.00000878	0.00000800	D(0.031)	D(0.953)	D(1)	D (0.942)	P
c.818C>T(p.T273I)	0.00001653	0.00001594	D(0)	D(0.999)	D(1)	D (0.759)	LP
c.1134C>G(p.D378E)	—	—	D (0.021)	D(0.663)	D(1)	D (0.787)	VUS
c.1006G>A(p.G336S)	—	0.00000398	D (0.026)	D(1)	D(1)	D (0.917)	LP

D, Damaging; P, Pathogenic; LP, Likely pathogenic; VUS, Uncertain significance.

**FIGURE 2:**
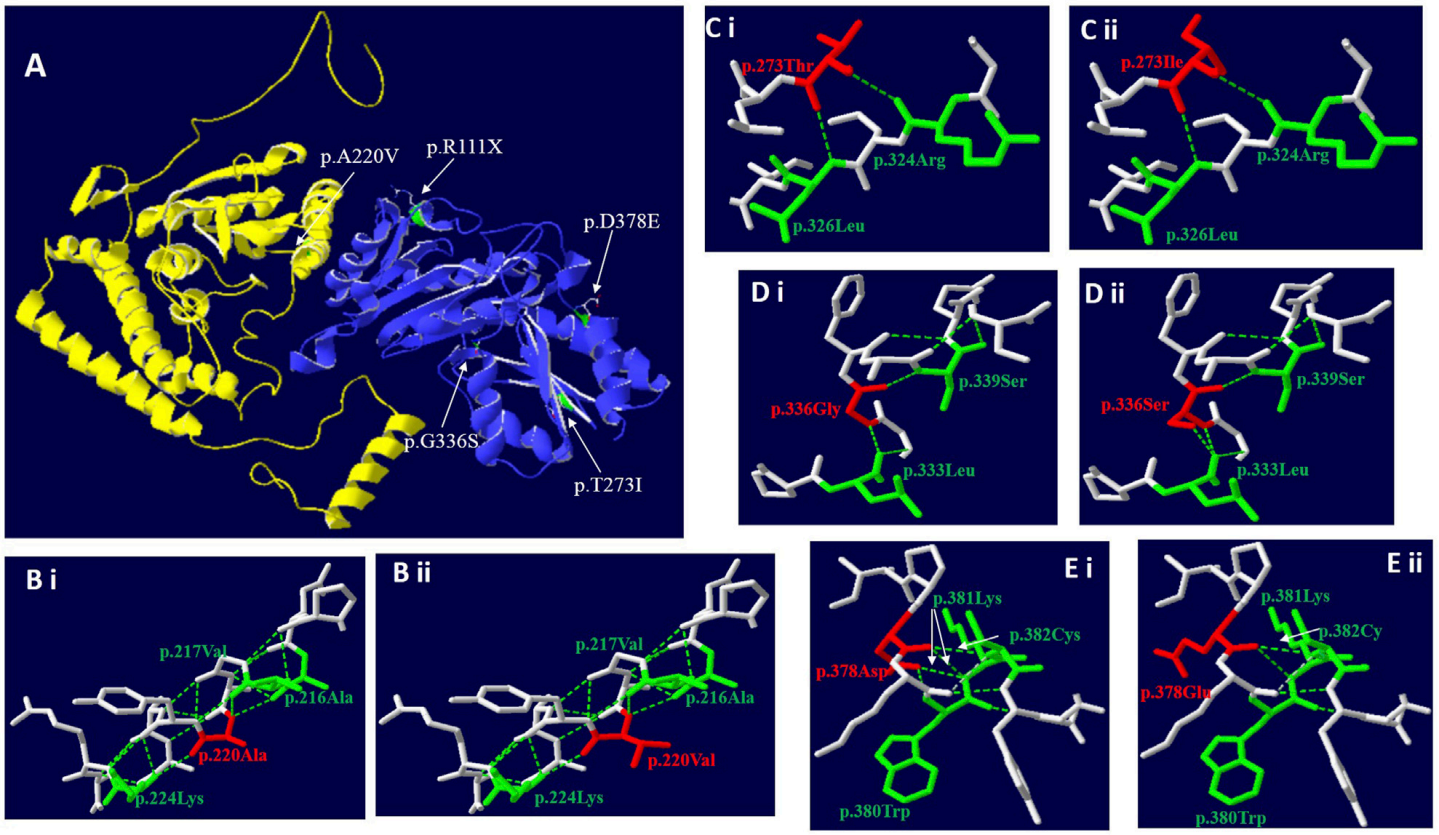
Three-dimensional structure of BCKDHA and BCKDHB (wild-type and mutant). **(A)** Overall structure of the BCKD protein. The chain α was highlighted in yellow, while chain β in blue. The location of the missense mutations were mapped to the regions of the protein in green and marked with white arrows. **(B)** The p. A220V missense mutation didn’t affect hydrogen bond linkage with adjacent amino acids, but changes the side chain structure. **(C)** The p.T273I missense mutation didn’t affect hydrogen bond linkage with R324 and L326, but changes the side chain structure. **(D)** The p.G336S missense mutation could create a new hydrogen bond between S336 and the Leucine (Leu) at position L333. **(E)** The p. D378E missense mutation could disrupt a hydrogen bond between E378 and K381 and between E378 and W380, respectively.

Use Swiss PBD software to predict the effect of missense mutation on protein conformation on BCKDHA and BCKDHB respectively (Figure 2A):

c. 659C>T (p.A220V): In the three-dimensional structure of human BCKDHA protein, alanine at position 220 respectively forms hydrogen bonds with alanine at position 216, valine at position 217, and lysine at position 224 (Figure 2Bi). The variant c.659C>T in *BCKDHA* resulted in alanine at position 220 becoming valine. The amino acid change does not affect the hydrogen bond linkage with adjacent amino acids, but changes the side chain structure (Figure 2Bi).

c.818 C > T (p.T273I): In the three-dimensional structure of human BCKDHB protein, threonine at position 273 respectively forms hydrogen bonds with arginine at position 324 and leucine at position 326 (Figure 2Ci). The variant c.818C>T in *BCKDHB* resulted in the replacement of polar threonine at position 273 by non-polar isoleucine. The amino acid change does not affect the hydrogen bond linkage with adjacent amino acids but changes the side chain structure (Figure 2Cii).

c.1006G > A (p.G336S): In the three-dimensional structure of human BCKDHB protein, glycine at position 336 respectively forms hydrogen bonds with leucine at position 333 and serine at position 339(Figure 2Di). The variant c.1006G > A leads to the substitution of glycine at 336 position by serine, and the change of amino acid leads to an additional hydrogen bond with leucine at 333 position, which is expected to affect the stability of protein structure (Figure 2Dii).

c. 1134C > G (D378E): In the three-dimensional structure of human BCKDHB protein, aspartic acid at position 378 respectively formed hydrogen bonds with tryptophan at position380, lysine at position 381 and cysteine at position 382 (Figure 2Ei). The variant c.1134C>G in *BCKDHB* resulted in glutamic acid replacing aspartic acid at position 378. The change of amino acid makes it lose one hydrogen bond with leucine 380 and lysine 381 respectively, which is expected to affect the stability of protein structure (Figure 2Eii).

## Discussion

In this study six patients with MSUD were diagnosed, thecase 1 was Intermediate MSUD, and the other 5 cases were Classic, which developed within the first two weeks after birth. The study used high-throughput sequencing to clarify the genetic etiology of the six patients (Table 1). All the patient have mutations in BCKDC subunit, diagnosing diseases from molecular etiology can improve the efficiency of disease diagnosis. High-throughput sequencing is essential for the MSUD’s differential diagnosis, early treatment, and prenatal diagnosis.

BCKDC is a highly conserved and catalytic complex essential for a series of enzymatic reactions, including branched-chain α-keto acid decarboxylase (E1), branched-chain acyltransferase (E2), dihydrolipoyl dehydrogenase (E3), BCKD kinase and BCKD phosphatase (Zeynalzadeh et al., 2018; Strauss et al., 2020; Sun et al., 2020). Mutations in any BCKDC subunits or enzyme protein genes can affect their catabolic functions. E1 is a tetramer composed of two E1α and two E1β. They are encoded by the *BCKDHA* (OMIM 608348) and *BCKDHB* (OMIM 248611) gene respectively. The human *BCKDHA* gene is located at 19q13.2, which contains 9 exons and 8 introns with a full gene length of 27 kb and encodes 445 amino acids. The human *BCKDHB* gene is located at 6q14.1, which contains 10 exons and 9 introns with a full length of 239 kb and encodes 392 amino acids. Patients with *BCKDHA* and *BCKDHB* gene mutations tend to have a Classic MUSD clinical manifestation (Nguyen et al., 2020; Jiang et al., 2021). E2 is encoded by the *DBT* (OMIM 248610) gene. The human *DBT* gene is located at 1p21, which contains 11 exons and 10 introns. The full length of *DBT* is 62 kb and encodes 482 amino acids. E3 is the homodimer of E2, encoded by the *DLD* (OMIM 238331) gene and located in 7q31.1, containing 13 exons and 12 introns. *DLD* is 28 kb in length, encodes 486 amino acids, and encodes a specific kinase. The *PPM1K* (OMIM 611065) gene, located at 4q22.1, contains seven exons and six introns. The full length of *PPM1K* is 26 kb long and encodes 372 amino acids. *PPM1K* encodes mitochondrial protein phosphatase that also causes MSUD when gene mutated (Nguyen et al., 2020). As of February 2022, the HGMD database (http://www.biopku.org/pnddb/search-results.asp) has included 439 mutations on BCKDH, of which *BCKDHA* accounts for 30% (132/439), *BCKDHB* for 39.4% (173/439), *DBT* for 23.5% (103/439), *DLD* for 6.8% (30/439), and *PPM1K* for 0.2% (1/439). The mutations were in various forms, including missense mutations, non-sense mutations, splice mutations, and shift mutations, among which missense mutations accounted for the most significant proportion.

Case 1 carried the compound heterozygous mutations c.659C>T (p.A220V) and c.795delG (p.N266Tfs*64) in the *BCKDHA* gene (Figure 1A). Case 2 was a patient confirmed by neonatal screening. Gene test revealed that the patient carried the *BCKDHB* gene complex heterozygous mutation c.818C>T(p.T273I) and c.1134C>G(p.D378E) (Figure 1B). Case 3 was in the neonatal period and carried the *BCKDHB* gene compound heterozygous mutations c.550delT (p.S184Pfs*46) and c.718delC (p.P240Lfs*14) (Figure 1C). Case 4 carried *BCKDHB* gene compound heterozygous mutations c.331C>T (p.R111*) and c.1006G>A (p.G336S) (Figure 1D). Gene detection of case 5 and case 6 revealed that the patient carried a homozygous mutation of *DBT* gene c.1291C>T (p.R431*) (Figures 1E, F) and was accompanied by mass spectrum abnormality (Table 2). Among the six patients diagnosed, 66.7% were male, two-thirds were Han nationality and one-third of them were Hui nationality. Three patients carried the *BCKDHB* gene mutation, two carried the *DBT* gene mutation, and one carried the *BCKDHA* gene mutation. With a total of nine mutations, including four missense mutations: c.659C>T (p.A220V), c.818C>T (p.T273I), c.1134C>G (p.D378E), and c.1006G>A (p.G336S); two non-sense mutations: c.1291C>T (p.R431*) and c.331C>T (p.R111*); three deletion mutations: c.550delT (p.S184Pfs*46), c.718delC (p.P240Lfs*14) and c.795delG (p.N266Tfs*64). All mutations were reported variants except c.1134C>G (p.D378E) and c.718delC (p.P240Lfs*14). The deletion mutation c.718delC (p.P240Lfs*14) leads to early termination of protein coding, with clear strong pathogenicity. Analysis of the missense mutations by bioinformatics software indicated that the mutations were strongly pathogenic. In order to evaluate the influence of missense mutation on protein function, we used SWISS software to analyze the protein 3D structure of missense variants. The results revealed that c.659C>T(p.A220V) and c.818C>T(p.T273I) lead to changes in the structure of the side chain (Figure 2Bi&Ci),c.1006G>A(p.G336S) and c.1134C>G(p.D378E) resulted in changes in the number of bonded hydrogen bonds (Figure 2Di&Ei), which affects the protein conformation of *BCKDHA* and *BCKDHB*. The discovery of new mutations not only enriches the genetic mutation spectrum of the disease, but also can diagnose the disease from molecular etiology as early as possible, improve the efficiency of disease diagnosis, achieve early detection, early diagnosis and early treatment, and provide effective genetic counseling and prenatal diagnosis for the patient’s family.

Similar to previous research reports, as it is a rare disease and the cases in each study are relatively limited, so the relationship between MSUD genotype and phenotype cannot be determined. Generally, most patients with *BCKDHA* and *BCKDHB* gene mutations are classical, with BCKDH activity less than 2% (Mitsubuchi et al., 2005; Fang et al., 2021). In our study, three patients with *BCKDHB* mutations are Classic, and one patient with *BCKDHA* mutations Intermediate MSUD. According to the literature, the mutation c.659 C>T is associated with moderate phenotype (Couce et al., 2015), we suspect that this may be the cause of mild clinical phenotype in case 1. The clinical manifestations of patients with *DBT* gene variant are relatively mild, but in this study, two classical patients who carried *DBT* homozygous mutations, both with neonatal onset and death, were classic MUSD patients. A previous study reported that a classical patient with the c.1291C>T (p.R431*) homozygous mutation died at six months (Khalifa et al., 2020). The report indicated that another patient who carried the c.1291C>T (p.R431*) homozygous mutation was diagnosed four days after birth with feeding difficulties and seizure symptoms, diagnosed as MSUD (Abiri et al., 2019). Mutation c.1291C>T (p.R431*) results in considerably impaired enzyme activity. According to the reported cases, c.659C>T(p.A220V) mostly appeared in compound heterozygous mutations and was reported as an Intermediate MSUD (Rodríguez-Pombo et al., 2006). Therefore, this study inferred that the enzyme activity of c.659C>T(p.A220V) mutation is less impaired. Among the reported cases, the four reported variations c.818C>T (p.T273I), c.1006G>A (p.G336S), c.331C>T (p.R111*), and c.550delT (p.S184Pfs*46) appeared in the form of compound heterozygous, these mutations would cause classic MSUD patients (Ali and Ngu, 2018; Li et al., 2018; Yang et al., 2019). These results indicated that the mentioned four reported mutations profoundly influenced the enzyme activity.

MSUD is a highly lethal and disabled genetic metabolic disease, and early diagnosis and treatment can vastly enhance the prognosis process. However, because of the lack of specific clinical manifestations in early MSUD, unscreened newborns with MSUD are highly susceptible to misdiagnosis as sepsis, neurological disorders, and other disorders that cause convulsions (Han et al., 2018; Liu et al., 2018). Therefore, making a diagnosis mainly based on clinical manifestations is difficult, which may lead to delayed treatment of misdiagnosed children. Thus, combining conventional differential diagnosis with high-throughput sequencing technology to explore the genetic etiology of MSUD patients and establish genotypic and phenotypic correlations becomes essential for rapid diagnosis of MSUD. Early symptomatic intervention and prenatal diagnosis of the patient become necessary.

## Conclusion

In conclusion, this study conducted a detailed analysis of the clinical conditions and gene mutations of six cases of MSUD. Two new pathogenic mutations of the *BCKDHB* gene were found through high-throughput sequencing, which enriched the mutation spectrum of the *BCKDHB* gene. The association analysis of genotype and phenotype greatly assisted clinical diagnosis and treatment. This study suggests that clinicians should pay more awareness to genetic testing and use high-throughput sequencing technology to identify the etiology rapidly and enhance disease diagnosis efficiency. It would enable early diagnosis and timely and effective treatment of MSUD patients and provide effective genetic counseling and prenatal diagnosis for their families.

## Data Availability

The original contributions presented in the study are publicly available. This data can be found here: https://www.ncbi.nlm.nih.gov/search/all/?term=SRR22979157-60.
