# Supplementary material for: Identification of gene mutations in six Chinese patients with maple syrup urine disease
Source: Front Genet. 2023 Feb 24;14:1132364. doi: 10.3389/fgene.2023.1132364 (PMC10001893; doi:10.3389/fgene.2023.1132364)
Supplement: Supplementary file 1 [file Table1.DOCX]

**Supplementary Table 1. Primers used in this study.**

| **Primer name** | **Sequence (5' → 3')** | **T_m_ (°C)** | **Size (bp)** |
| --- | --- | --- | --- |
| *BCKDHA-*F1 | AATCCTGCCACCTTCCTGC | 60 | 470 |
| *BCKDHA-*R1 | AAAGGCAAGGGGGAGATGC |  |  |
| *BCKDHB-*F1 | TGGTAACTGTCATCCAGTGGG | 60 | 439 |
| *BCKDHB-*R1 | AGGAATTCATCCATACAATGGG |  |  |
| *BCKDHB-*F2 | GAAGGAAGGAAGGGAGGGAG | 60 | 250 |
| *BCKDHB-*R2 | CCTTGATTCCTGGGCAATGG |  |  |
| *BCKDHB-*F3 | GCAGTAATGTCATGGAGCCA | 60 | 674 |
| *BCKDHB-*R3 | TCCGTACCACAGACTTTCAGT |  |  |
| *BCKDHB-*F4 | CCTGTCGAAAGCGAGTTGTAA | 60 | 375 |
| *BCKDHB-*R4 | GAAAGCATGCACTACATTTCCAT |  |  |
| *BCKDHB-*F5 | CTGCAGGAGGAATGTTTCTTG | 60 | 232 |
| *BCKDHB-*R5 | CTGATGATTGCTGTGTCTTGG |  |  |
| *DBT-F1* | CACAAATTGGAGAAGCGTGA | 60 | 231 |
| *DBT-*R1 | GGAAGAAGGGTTTGCCTGAT |  |  |
